# Transcriptome profiling of pluripotent pig embryonic stem cells originating from uni- and biparental embryos

**DOI:** 10.1186/s13104-020-04987-6

**Published:** 2020-08-03

**Authors:** Kwang-Hwan Choi, Dong-Kyung Lee, Jong-Nam Oh, Seung-Hun Kim, Mingyun Lee, Sung Woo Kim, Chang-Kyu Lee

**Affiliations:** 1 Department of Agricultural Biotechnology, Animal Biotechnology Major, and Research Institute of Agriculture and Life Science, Seoul National University, Seoul, 08826 South Korea; 2 Animal Genetic Resources Research Center, National Institute of Animal Science, RDA, Namwon, Jeollabuk-do 55717 South Korea; 3 Institute of Green Bio Science and Technology, Seoul National University, Pyeong Chang, Kangwon-do 25354 South Korea

**Keywords:** Pig embryonic stem cells, RNA-seq, Transcriptome, In vitro fertilized embryo, Parthenogenetic embryo, Preimplantation embryo

## Abstract

**Objectives:**

Pig pluripotent stem cells have tremendous potential because the pig is a valuable animal as both an agricultural resource and as a preclinical model of human therapy. To date, a lack of understanding of pig pluripotency has inhibited the derivation of embryonic stem cells (ESCs) and transgene-free induced pluripotent stem cells. Therefore, there has been no accessible or reliable transcriptome data for researching the genuine pig pluripotency network. Our previous study isolated authentic pig ESCs, which had teratoma-forming and direct differentiation ability, that were derived by activating the FGF2, ACTIVIN A, and WNT pathways. Here, we aimed to provide detailed information on transcriptome data of the newly derived pig ESCs and perform a comparative analysis with pig preimplantation embryo transcriptomes in a public database.

**Data description:**

The transcriptome data of ESCs derived from in vitro fertilized and parthenogenetic embryos were generated by HiSeq 2500. Then, differentially expressed genes (DEGs) from each sample were compared with fibroblasts, and gene expression profiling was carried out for comparative analysis. Our data, as the first transcriptome dataset for genuine pig pluripotent cells, could be a general reference for explaining the molecular mechanism of species-specific pluripotency and improving understanding of the embryo development of domestic animals.

## Objective

The pig is a valuable animal as a preclinical model of human therapy as well as an agricultural resource. Recent studies have found that pig germ cell development more closely resembles the molecular features of human germ cell specification than that of the mouse [1] and that the pig is an ideal candidate for interspecies-chimera formation with human pluripotent stem cells (PSCs) to provide a source for xenotransplantation of organs [2]. Accordingly, research into pig PSCs such as embryonic stem cells (ESCs) and induced pluripotent stem cells (iPSCs) is required for understanding their comparative developmental biology and medical/agricultural applications. However, to date, no stable and well-characterized pig PSCs have been derived due to a lack of understanding of pig pluripotency networks and suitable culture conditions [3, 4]. Instead, ES-like cells, having no in vivo differentiation ability, and transgene-dependent partially reprogrammed iPSCs have been derived and researched over the last few decades [5]. Recently, through systemic screening of key pathways for pig pluripotency circuitry, two independent research groups, including our group, reported two different types of genuine pig PSCs derived from embryos that have in vivo and in vitro differentiation potentials [6, 7].

Transcriptome data of PSCs derived from embryos and somatic cells are considered to be valuable tools for the investigation of pluripotency. Transcriptome analysis of pluripotent cells provides a comprehensive biological insight into developmental cues that we couldn’t acquire before through traditional gene analysis techniques. Pluripotent cells originate during the preimplantation period of embryo development and the reprogramming process of somatic cells [8]. Accordingly, this study aimed to offer comprehensive information and improve the reusability of an RNA-sequencing (seq) data set of pig ESCs derived from in vitro-fertilized and parthenogenetic embryos.

## Data description

### Cell preparation and RNA extraction

Pig ESCs were cultured with feeder cells made of mitotically inactivated mouse embryonic fibroblasts to maintain their pluripotency in vitro. Therefore, the feeder cells needed to be removed from the ESC samples to obtain a highly reliable RNA-seq data from a homogenous population of the undifferentiated pig ESCs. To exclude these contaminating cells, the pig ESCs were sorted by a MACS system using an antibody against SSEA1, which is highly expressed on the surface of undifferentiated pig ESCs [6]. The RNA integrity number (RIN) and 28S/18S rRNA ratio were determined by the Agilent 2100 BioAnalyzer to estimate the integrity and degradation level of the extracted RNA samples. The RIN values and 28S/18S rRNA ratio of the samples used in this study were greater than 9.2 and 1.9, respectively, which indicated that the samples for RNA-seq have high integrity and a low degradation level (Table S1, Table 1; All of the data files described in this data note is summarized in Table 1).

**Table 1:** Overview of data files/data sets

Label	Name of data file/data set	File types (file extension)	Data repository and identifier (DOI or Accession Number)
Supplementary file 1	Methodology description	Document file (.pdf)	Figshare (10.6084/m9.figshare.10298324.v2)
Dataset 1	Raw FASTQ files for the RNA-seq. of pig embryonic stem cells	Fastq files (.fastq)	NCBI Gene Expression Omnibus (GSE120031)
Dataset 2	List of normalized gene expression level (FPKM; ESCs and Embryos)	Spreadsheet (.xlsx)	Figshare (10.6084/m9.figshare.10298324.v2)
Dataset 3	Lists of differentially expressed genes (FC; ESCs and Embryos)	Spreadsheet (.xlsx)	Figshare (10.6084/m9.figshare.10298324.v2)
Table S1	Summary of cell samples for RNA-seq.	Spreadsheet (.xlsx)	Figshare (10.6084/m9.figshare.10298324.v2)
Table S2	Summary of alignment statistics for RNA-seq.	Spreadsheet (.xlsx)	Figshare (10.6084/m9.figshare.10298324.v2)
Table S3	Number of differentially expressed genes (DEGs) compared with fibroblasts	Spreadsheet (.xlsx)	Figshare (10.6084/m9.figshare.10298324.v2)
Figure S1	Quality assessment of raw FASTQ files	Document file (.pdf)	Figshare (10.6084/m9.figshare.10298324.v2)
Figure S2	Workflow of the transcriptome analysis and the results of comparative analysis	Document file (.pdf)	Figshare (10.6084/m9.figshare.10298324.v2)
Figure S3	Gene expression profiling in pig ESC lines and preimplantation embryos	Document file (.pdf)	Figshare ( 10.6084/m9.figshare.10298324.v2)

### Quality control assessment

As evaluated by the Agilent 2100 BioAnalyzer, all of the libraries used in this study have a uniform and constant size of approximately 380 bp and passed quality control to be used for sequencing. Then, base call quality scores were calculated by the FastQC program to assess the quality of the raw sequencing data of the cDNA libraries (Dataset 1). Low-quality reads were filtered according to the criteria as described in the Methodology (Supplementary file 1). After filtration of low-quality reads, as shown in the box plot distributions of Figure S1, the quality scores across all bases were in the high confidence range. The filtered sequence reads and previously reported data for pig preimplantation embryos [9] were aligned to the pig reference genome (Sscrofa10.2) with 80.5–92.2% mapping rates (Table S2). After the mapping, gene expression levels were calculated by the HTSeq-count program (Fig. S2a, Dataset 2). These data were visualized by the R program as an MDS plot, which demonstrated four clusters including fibroblasts, ESCs, epiblasts and inner cell mass (ICM) (Fig. S2b).

### Comparative analysis of gene expression

The R package TTC was used for differentially expressed genes (DEGs) analysis (Dataset 3). DEGs of embryos and ESCs compared with fibroblasts were measured based on cutoffs of fold change > 2.5 and a *p* value < 0.05 (Table S3 and Fig. S2c). Compared with fibroblasts, 2189, 2450 and 2327 genes were upregulated in PG-ES-3, PG-ES-7 and IVF-ES-11, respectively, and 1887 genes were commonly expressed in the three different ESC lines (Fig. S2d). Moreover, upregulated DEGs in the embryo samples were compared with genes commonly upregulated in ESCs. As a result, 200 genes were commonly expressed in all samples, and 936, 542, 269 and 283 genes were uniquely expressed in ESCs, day 7–8 ICM, day 10–11 epiblasts and day 12–13 epiblasts, respectively (Fig. S2e). Finally, expression of genes related to pluripotency, cellular signaling and lipid metabolism were analyzed (Fig. S3). Pig ESCs had more similarity with epiblasts than the ICM, indicating similar patterns observed in a previous study [6].

## Limitations

The sample size of the experimental groups was not sufficient for reliable comparative analysis. Gender and sequencing platforms were not matched between ESCs and embryo samples.

## Data Availability

Raw FASTQ files for the RNA-seq were deposited in the GEO database under accession number GSE120031 (https://identifiers.org/geo:GSE120031) [10]. The methodology description and processed data (dataset 1, dataset 2, Tables S1–S3, and Figures S1–S3) are available in Figshare (10.6084/m9.figshare.10298324.v2) [11].

## Abbreviations

PSCs: Pluripotent stem cells
ESCs: Embryonic stem cells
iPSCs: Induced pluripotent stem cells
ICM: Inner cell mass
DEGs: Differentially expressed genes
RIN: RNA integrity number
RNA-seq: RNA-sequencing
